# Insect-Specific Flaviviruses Have Potential Applications as a Scaffold for Pathogenic Flavivirus Vaccines

**DOI:** 10.3390/vaccines13070769

**Published:** 2025-07-21

**Authors:** Jia-Zhen Cui, Xiang-Hua Xiong, Qing-Yang Wang, Hao-Long Dong, Gang Liu, Hui-Peng Chen

**Affiliations:** Academy of Military Medical Sciences, Beijing 100850, China

**Keywords:** insect-specific flavivirus, vaccine scaffold, pathogenic flavivirus, vaccine development, host restriction

## Abstract

Pathogenic flaviviruses are predominantly the pathogens of emerging and re-emerging infectious diseases, which have caused multiple public health emergencies globally and pose a serious threat to human health and social development. Although significant achievements have been made in vaccine research, issues such as limited protective effects and virulence reversion persist, making the development of novel vaccines against pathogenic flaviviruses a current research hotspot and challenge. ISFVs have recently attracted attention due to their high homology with pathogenic flaviviruses and unique inability to replicate in mammalian hosts. Multiple vaccine candidate strains constructed using ISFVs as scaffolds have demonstrated excellent safety and efficacy. This review summarizes the biological characteristics, host restriction factors, current applications in vaccine development, and challenges faced by ISFVs, providing a reference for future research on pathogenic flavivirus vaccines.

## 1. Introduction

Pathogenic *orthoflavivirus* (also known as pathogenic flavivirus) is widely distributed and poses a consistent threat to human health, imposing a significant burden on the global economy [1,2]. In recent years, climate change, urbanization processes, and cross-border travel have accelerated the expansion of mosquito-borne infectious diseases, leading to frequent outbreaks of flavivirus-related epidemics [3,4,5]. Despite clinical approval of vaccines for some flaviviruses [6,7], live attenuated vaccines carry risks such as virulence reversion and low safety [8]; inactivated vaccines offer limited protective effects, making it difficult to achieve long-term protection even with multiple immunizations [9]. Thus developing a safe and efficient novel vaccine platform is urgently needed.

In recent years, a unique class of insect-specific flaviviruses (ISFVs) has provided new insights for vaccine development due to their high homology with pathogenic flavivirus genomes and inherent host restriction characteristics [10]. ISFVs complete their replication cycle only in arthropod cells such as mosquitoes and cannot establish effective infection in mammalian cells. This characteristic not only avoids the risk of virulence reversion associated with traditional live-attenuated vaccines but also confers a natural safety advantage as a viral vector or antigen presentation system. Multiple chimeric flavivirus vaccine candidate strains constructed using ISFVs have demonstrated good safety and efficacy at the animal level, and this vaccine development platform is expected to provide certain reference value and application significance for the prevention and control of pathogenic flaviviruses [11,12].

## 2. Pathogenic Flavivirus Severely Impacts Human Health

The *orthoflavivirus* genus (Flavivirus genus) includes vertebrate infectious flaviviruses (VIFs), ISFVs, and viruses with undetermined transmission vectors [13,14]. Among these, multiple viruses within VIFs pose serious threats to human health; they are major pathogenic flaviviruses and include Dengue virus (DENV), Yellow Fever virus (YFV), Zika virus (ZIKV), West Nile virus (WNV), and Japanese encephalitis virus (JEV), etc. These viruses are transmitted by arthropods such as mosquitoes or ticks [15], causing a heavy disease burden worldwide, infecting over 400 million people each year and triggering millions of severe cases [16,17].

Pathogenic flavivirus infections present a broad spectrum of clinical manifestations, ranging from self-limiting fever to fatality, which can be roughly divided into two phenotypes: systemic diseases involving hemorrhage and neurosystem-related complications [18]. DENV can progress to dengue hemorrhagic fever and shock syndrome. Severe neurological infections (such as encephalitis) are caused by WNV, JEV, and tick-borne encephalitis virus (TBEV) [19,20]; YFV infection can lead to fatal visceral disease; ZIKV is associated with congenital defects such as microcephaly in newborns. Although the pathogenic mechanisms of different viruses vary, their common features include high incidence, risk of severe illness and global spread trend. Taking ZIKV as an example, an outbreak occurred across the South Pacific in 1997; subsequently, it spread to the Americas with a major epidemic, causing over half a million suspected cases in South America between 2015 and 2017, and from February to November 2016, the WHO declared the ZIKV epidemic a Public Health Emergency of International Concern [21,22]. Due to urbanization and globalization progression, as well as climate change, pathogenic flaviviruses are spreading globally and expanding geographically [23].

Pathogenic flaviviruses impose a huge burden on global health; yet most viruses still lack clinically approved vaccines. Common vaccines for pathogenic flaviviruses include inactivated vaccines, live attenuated vaccines, nucleic acid vaccines, and subunit vaccines. These traditional vaccines have problems such as long development cycles, insufficient immunogenicity, or potential risks of virulence recovery [24]. For instance, live attenuated vaccines may have the potential risk of virulence atavism due to random mutations, recombination, or evolutionary adaptation, resulting in low safety, while inactivated vaccines generally have limited protective effects, etc. [25,26]. The above reasons are also a major cause of the relatively small number of pathogenic flavivirus vaccines approved in clinical practice to date. At present, a few virus vaccines, such as YF-17D [27] for YFV, the QDenga vaccine [28], the Dengvaxia/CYD-TDV vaccine [29] for DENV, SA14-14-2 [30], and IMOJEV/THAIJEV [31] for JEV, have been approved for clinical use. YF-17D is a live attenuated vaccine obtained through continuous passage [32], demonstrating excellent safety and immunogenicity and marking a milestone in the history of vaccine research and development. Although YF-17D has a long history of clinical application, there have been reports of adverse reactions such as vaccine-related visceral and neurological diseases in people with immunodeficiency and in the elderly [33,34,35,36]. Both of the DENV vaccines, QDenga and Dengvaxia/CYD-TDV, are chimeric attenuated live vaccines, targeting different indications. QDenga can be administered to both seropositive and seronegative individuals, while Dengvaxia is only recommended for seropositive individuals and contraindicated in seronegative individuals. There are four serotypes of the DENV virus (DENV1-DENV4), and their antigenic structures are similar but have differences. Antibodies produced against a certain serotype may have only partial cross-neutralizing ability against other serotypes, or even no neutralizing ability at all, and instead enhance infection through the antibody-dependent enhancement (ADE) mechanism. This is a difficulty in the development of DENV virus-related vaccines. Among the two approved vaccines for the JEV virus, SA14-14-2 is a live attenuated vaccine obtained through continuous multiple passages of the wild-type JEV SA14 strain (Genotype III, GIII). It provides complete protection against the GIII strain but is only partially effective against other genotypes such as GI and GV. Its attenuated mechanism involves mutations at key sites of the E protein [37]. In recent years, the prevalence of the JEV virus has gradually shown a trend of GI type replacing GIII type. SA14-14-2 cannot provide complete protection against the newly prevalent subtype of JEV. Among the above-mentioned vaccines, both DENV (Dengvaxia/CYD-TDV) and JEV (IMOJEV/THAIJEV) are structural protein gene (prME) chimeric vaccines constructed with the attenuated live vaccine YF-17D as the scaffold. The YF-17D scaffold chimeric vaccine retains the safety of the attenuated parent virus of YF-17D while demonstrating good immune efficacy [38,39]. Chimeric vaccines constructed with YF-17D as the scaffold also have risks such as unknown attenuating principles of the vaccine and possible virulence reversion. The above-mentioned vaccines have been widely applied and have played a significant role in the prevention and control of pathogenic flaviviruses, suggesting that effective vaccine strains can be constructed by modifying the genomic structure of flaviviruses and through continuous passage and other methods. The sustained efficacy, safety, and supply of these vaccines remain a major issue. In addition, vaccines targeting pregnant women as the main immune group, such as ZIKV vaccines, have even higher requirements for the safety and effectiveness of the vaccines. With the current vaccine research and development technologies, it is difficult to solve the corresponding problems. There is an urgent need for new technologies that can solve these problems and achieve safe and economical vaccine production in high-demand countries.

## 3. Biological Characteristics of ISFVs

ISFVs (ISFVs), belonging to the Flavivirus genus, were previously overlooked due to their exclusive infection of arthropods, but have been widely reported by the scientific community in recent years. The first discovered insect-specific flavivirus was cell fusing agent virus (CFAV), isolated from Aedes aegypti cell cultures [40,41]. Subsequently, ISFVs were isolated worldwide and classified into two lineages: Lineage I (classical, cISF) and Lineage II (dual-host associated dISF) [42]; Lineage II is also known as dual-host affiliated ISFVs [43]. ISFVs have a global distribution with geographical specificity, primarily existing in tropical and subtropical regions, with distribution overlapping mosquito habitats, mainly infecting arthropods such as mosquitoes. ISFVs share highly similar genome size and structure with pathogenic flaviviruses, with a genome size of approximately 10–11 kb, encoding a single ORF that produces three structural proteins (C, prM, E) and seven non-structural proteins (NS1, NS2a, NS2b, NS3, NS4a, NS4b, NS5), where prME serves as the primary viral antigen, and the 5′ and 3′ untranslated regions (UTRs) possess conserved secondary structures. The virus primarily propagates through vertical transmission (transovarial transmission) amongst arthropods and does not transmit to vertebrates via blood feeding [44,45,46]. ISFVs differ significantly from pathogenic flaviviruses in host range, replication capacity, pathogenicity, and transmission routes (Table 1): they exclusively infect arthropod cells, remaining non-infectious to mammalian cells, unlike pathogenic flaviviruses that can establish infection in both mosquito and mammalian cells. ISFVs are completely unable to replicate in mammalian cells and exhibit no pathogenicity to mammals, making them an ideal scaffold for pathogenic flavivirus vaccines.

## 4. Host Restriction Factors of ISFVs

ISFVs, as a unique group within the Flavivirus genus, are characterized by their strict host restriction towards vertebrate cells. This host limitation occurs at multiple stages of the viral life cycle, including entry and replication [46,47,48,49], and the virus’s inability to infect vertebrate cells is determined by a combination of viral structural characteristics and host–virus interaction mechanisms.

### 4.1. Viral Characteristic Limitations

The genomic and structural characteristics of the virus itself are important factors in determining its host range. These features include elements such as UTRs, capsid proteins, and non-structural proteins. Viral UTRs play a significant role in the host range. The UTR of the flavivirus genome is a highly conserved untranslated region that interacts with viral and host proteins during viral replication, influencing key steps such as translation, replication, and encapsidation [50]. The research reports that the untranslated region of the virus is a key obstacle to the host restriction of ISFVs in vertebrate cells. The insect-specific flavivirus Donggang virus (DONV) could not replicate in vertebrate cells. After replacing the UTRs of the Donggang virus with that of ZIKV, the Donggang virus could replicate in vertebrate cells [51]. When the UTRs and capsid protein of insect-specific flavivirus were replaced with ZIKV, the chimeric ZIKV could not infect vertebrate cells [52]. The above research results indicate that the UTRs are a key factor in host restriction. In addition, viral non-structural proteins include different enzymes and cofactors required to complete the replication cycle in the infected host, which are believed to affect the host range of ISFVs [53].

### 4.2. Host–Virus Interactions Limitation

Viral tropism, which is the ability of a virus to effectively infect and replicate in specific cell types, is the basis of host restriction in ISFVs and influences viral infection and disease progression [54]. Viral tropism is determined by host cell susceptibility, including receptor binding and immune defense.

Vertebrate immune systems likely inhibited the replication of ISFVs. Although limited transcription of ISFV RNA occurs in IRF3^−/−^/IRF5^−/−^/IRF7^−/−^ murine fibroblasts, productive replication remains blocked, confirming vertebrate host restriction [55]. However, the RIG-I/MDA5 pathway present in vertebrate cells, which is absent in mosquito cells, may rapidly recognize dsRNA intermediates of ISFVs, activate interferon pathways, and limit viral replication. Zinc finger antiviral proteins (ZAPs) and others in vertebrate cells can bind to CpG dinucleotides in viral RNA, thereby preventing viral replication [56]. This might be one of the reasons why ISFVs cannot infect vertebrate cells. The absence of or variation in host factors such as AUF1 p45 and EF-1α in vertebrate cells might explain the observed inability of ISFVs to replicate [46]. Additionally, the significant temperature difference between ISFVs’ optimal replication temperature (25–28 °C) and mammalian homeothermic environments (37 °C) may suppress viral enzyme activity and affect viral replication [57].

In essence, the inability of ISFVs to infect vertebrate cells is the result of multiple factors working in concert. These multi-layered restriction mechanisms provide unique advantages for ISFVs as a vaccine development platform. Notably, ISFVs maintain characteristic flavivirus antigenic features, particularly in their prME protein architecture, which—combined with their inherent vertebrate replication deficiency—ensures vaccine vector safety while preserving antigenicity potential. Their inherent host restriction ensures the safety of vaccine vectors within vertebrate organisms, whilst retaining the characteristic immunogenicity typical of the Flavivirus genus. Current research is exploring ISFV modification strategies through reverse genetics techniques, with the aim of developing novel zoonotic vaccines

## 5. ISFVs Have the Potential to Be Used as a Scaffold for Pathogenic Flavivirus Vaccines

ISFVs and pathogenic flaviviruses share similarities in genome structure and replication mechanisms, making them an ideal model for studying flavivirus biology and developing novel vaccines. ISFVs can effectively replicate in arthropod vectors but cannot replicate in vertebrate hosts [58,59,60,61]. Given this characteristic, replacing the antigenic region (prME) of pathogenic flaviviruses with the corresponding region of ISFVs obtains a chimeric attenuated live vaccine candidate. The vaccine, subject to host restriction, retains the characteristic of ISFVs not infecting vertebrate cells. When administered at an appropriate dose, it will induce an effective immune response, stimulating the body to produce the corresponding antibodies and safely and efficiently achieving the purpose of vaccine strain stimulation.

**Figure 1:**
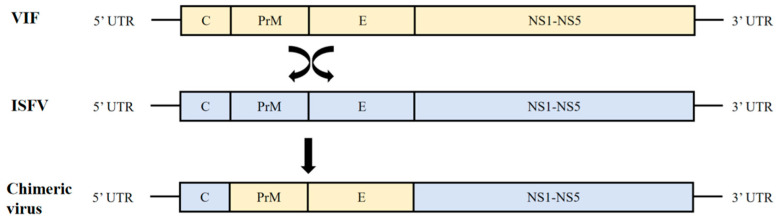
Chimeric virus construction strategy. Different colors represent different virus components.

Currently, multiple ISFVs have been reported as vaccine scaffold strains, such as Binjari virus (BinJV), Chaoyang virus (CYV/CHAOV), and Aripo virus (ARPV). The specific construction strategy is shown in Figure 1; vaccine candidate strains constructed using such viruses as a scaffold have demonstrated good safety and immunogenicity against pathogenic flaviviruses.

### 5.1. Constructing a Chimeric Flavivirus Vaccine Candidate Strain Using BinJV as the Scaffold

BinJV was isolated from *Aedes* normanensis in the Binjari community in Australia; it is a Lineage II insect-specific flavivirus [62]. BinJV replicates efficiently in C6/36 mosquito-derived cell lines, with replication deficiency in vertebrate cell lines, and this deficiency is not affected by temperature variations in the culture environment. Multiple studies have reported using circular polymerase extension reaction (CPER) to replace the BinJV prME gene with VIF’s prME gene, rescuing the chimeric BinJ/VIF prME virus [63]. The chimeric virus demonstrates strong replication in C6/36 cells whilst retaining replication deficiency in vertebrate cells, with immunofluorescence identifying chimeric virus expression of the corresponding VIF prME protein and high-resolution cryo-electron microscopy showing that the BinJ/VIF-prME chimera can fully express the corresponding VIF prME protein [16]. The chimeric virus demonstrates strong replication capacity in C6/36 cells while remaining replication-deficient in vertebrate cells, ensuring efficient production and attenuation for vaccine safety [64]. Multiple reports currently exist regarding Binjari virus-based chimeric flavivirus vaccine candidates, including BinJ/WN prME virus [65], BinJ/ZIKV prME virus [66], BinJ/DENV2 prME virus, BinJ/JEV prME virus, BinJ/YFV prME virus [67], BinJ/DENV1 prME virus, and BinJ/DENV4.

The chimeric flavivirus vaccine candidate based on the Binjari virus scaffold demonstrated good safety and efficacy, suggesting that the Binjari virus has potential as a universal scaffold for constructing vaccines against pathogenic flavivirus families. Taking the BinJ/ZIKV prME virus as an example, the vaccine candidate was proven to protect male and female IFNAR^−/−^ mice from viraemia without an adjuvant; protect male IFNAR^−/−^ mice from testicular damage; protect maternal and fetal IFNAR^−/−^ mice from ZIKV infection without inducing high-level neutralizing antibodies; and provide immunological protection for over 15 months [66].

### 5.2. Construction of a Chimeric Flavivirus Vaccine Candidate with CYV/CHAOV as the Scaffold

CYV/CHAOV is a newly discovered mosquito-borne flavivirus with replication deficiency in vertebrate cells [68]. The virus was isolated from mosquito samples in Chaoyang City, Liaoning Province, China, in 2008. RT-PCR detection and whole-genome sequencing analysis confirmed it as a subgroup II insect-specific flavivirus. CYV/CHAOV demonstrates efficient replication in mosquito cell lines (such as C6/36 cells). The virus can enter vertebrate cells but fails to initiate replication, exhibiting replication deficiency in vertebrate cells [51]. Similar to the Binjari virus, CYV/CHAOV may have potential as a vaccine scaffold. Through genetic engineering techniques (such as the CPER method), the prME gene of CYV/CHAOV can be replaced with prME genes from other pathogenic flaviviruses to construct chimeric viruses. There are reports of using the CPER method to construct a rescue chimeric ZIKV vaccine candidate ChinZIKV with CYV/CHAOV as the scaffold [69]. This chimeric vaccine could partially protect immunodeficient adult mice and suckling mice against the ZIKV virus at a vaccine dose of 10^4^ FFU without vaccine adjuvants, and ChinZIKV was completely safe in the experimental mouse groups [70].

### 5.3. Construction of a Chimeric Yellow Fever Vaccine Candidate Strain Using ARPV as the Scaffold

ARPV is a flavivirus of insect-specific Lineage II isolated from *Psorophora albipes* mosquitoes in Trinidad [71]. ARPV replication is confined to mosquito cells, with a notable cytopathic effect observed in mosquito cells. Although it cannot replicate in vertebrate systems, research demonstrates that ARPV is internalized into vertebrate cells via clathrin-mediated endocytosis and is highly immunomodulatory, capable of inducing a robust innate immune response in vertebrate systems even without replication.

Utilizing ARPV as a scaffold to construct the ZIKV virus chimeric vaccine strain Aripo-Zika (ARPV/ZIKV) demonstrated excellent safety, immunogenicity, and efficacy [72]. The vaccine construction strategy similarly involved replacing the prME region of the ZIKV virus with the corresponding region of the ARPV to obtain a chimeric virus vaccine. A near-linear relationship exists between the ARPV/ZIKV immunization dose and the protective effect, with 10^11^ genome copies (i.e., 10^8^ plaque-forming units) being the minimum dose to protect mice against ZIKV virus attack. The use of immunological adjuvants did not significantly improve the short-term therapeutic effect of the ARPV/ZIKV vaccine. Due to the inherent vertebrate host limitation of ARPV/ZIKV, the vaccine demonstrated outstanding safety. ARPV/ZIKV showed no replication or translation in vitro and no pathogenic action in vivo [73].

## 6. The Advantages and Disadvantages of Insect-Specific Flavivirus as the Scaffold of Pathogenic Flavivirus Vaccines

The greatest advantage of insect-specific flaviviruses (ISFVs) as a vaccine scaffold compared with traditional vaccines is their high safety. As ISFVs cannot replicate in humans, they avoid the risk of disease and provide a highly safe platform for vaccine development. Meanwhile, they can induce a powerful immune response and have demonstrated outstanding protective efficacy in preclinical studies. In addition, the ISFV scaffold has the potential for universality and can be used as an engineering platform [74] to rapidly prepare vaccine candidate strains against various pathogenic flaviviruses. Compared with traditional vaccine production methods such as attenuated live vaccines, it can achieve rapid and precise design and synthesis of vaccines. Secondly, chimeric vaccines can be produced in large quantities economically and efficiently in mosquito cells, providing a basis for the high demand for vaccines [69]. However, chimeric vaccines with ISFVs as the scaffold also have certain disadvantages. First, the precise molecular mechanisms underlying the vertebrate replication deficiency of ISFV-based chimeric vaccines have yet to be fully elucidated [75]; Secondly, the mechanism by which ISFV scaffold chimeric vaccines exert their immune effects remains unclear. The ISFV scaffold chimeric vaccine can only be obtained by amplification in mosquito cells, and it is difficult to avoid carrying mosquito antigens, which may increase the side effects of mosquito heterologous proteins during the vaccine application process. Finally, there is still a lack of effective data support for the long-term protective effect of ISFV scaffold chimeric vaccines.

## 7. Discussion and Prospects

Pathogenic flaviviruses, as important causes of emerging and re-emerging infectious diseases, have drawn global public health attention [63]. Although significant progress has been made in traditional vaccine development strategies, the potential risk of virulence atavism due to random mutations, recombination, or evolutionary adaptation in attenuated live vaccines, which leads to low safety, and the limited general protective effect of inactivated vaccines still restrict the vaccine development process [25,76]. In recent years, innovative strategies for constructing vaccine candidate strains based on ISFVs have demonstrated unique advantages: ISFVs naturally lack mammalian pathogenicity, fundamentally avoiding the potential virulence reversion risk of traditional attenuated strains [26]; their genomic structure is highly homologous with pathogenic flaviviruses, enabling rapid and precise replacement of key antigenic regions through chimeric techniques while preserving native conformational epitopes [69,72]; ISFVs can be efficiently produced in insect cells, facilitating large-scale vaccine production; multiple animal experiments have confirmed that ISFV-based vaccines can induce high-level neutralizing antibodies and cellular immune responses [73,77]. These characteristics provide a novel direction for developing safer universal flavivirus vaccine platforms.

In recent years, significant progress has been made in vaccine development based on ISFVs. Researchers have successfully inserted antigenic epitopes from various pathogenic flaviviruses into the ISFV genome, developing recombinant vaccines against multiple pathogenic flavivirus diseases. These chimeric vaccines have demonstrated immunogenicity and safety and have shown good protective effects in animal experiments, presenting extremely broad prospects for development and application. However, these studies remain in the preliminary stages and require larger-scale and longer-term clinical trials to comprehensively evaluate the vaccine’s efficacy and safety.

Although ISFVs have great potential as a vaccine skeleton, they still face multiple challenges. Firstly, the strict host restriction mechanism of ISFVs has not been fully elucidated, and the molecular basis of its transspecies barrier needs to be deeply analyzed through techniques such as reverse genetics. Secondly, although the replication defect of chimeric viruses in mammalian cells enhances safety, it may also limit the immunogenicity of the vaccine due to the antigenicity difference between ISFVs and pathogenic flaviviruses. Therefore, the protective effect of the vaccine needs to be further improved through rational design optimization or the development of new delivery systems or adjuvants. Furthermore, the cross-protective effects of different members of the Flavivirus genus still need to be systematically evaluated; in particular, the blocking effect in the vector-borne transmission link deserves attention. In addition, the development of vaccines based on ISFVs still faces deep-seated challenges: Although the strict host restrictions of ISFVs eliminate the risk of virulence atavism, they may weaken their immune activation efficacy in mammals. Most of the “high-level neutralizing antibodies” reported in the existing research are derived from short-term animal experiments. The effects of long-term memory T-cell response and mucosal immune protection have not been fully verified, and the low replication level caused by the host restriction mechanism may reduce the intensity of continuous antigen exposure. Although the ISFV skeleton itself may not directly cause ADE, the target antigens it displays (such as the domain of flavivirus E protein) still need to be carefully designed. The fusion loop (FL) epitope of flavivirus E protein is a key region mediating ADE. If the ISFV vaccine expresses chimeric proteins containing such epitopes, it may still induce cross-reactive antibodies, thereby posing a theoretical risk of ADE, which may restrict the future application of the vaccine [78,79].

Future research directions include the following: in-depth understanding of host restriction mechanisms of ISFVs to guide more rational vaccine design; strengthening optimization of ISFV vaccine production processes to meet large-scale vaccination requirements; focusing on constructing a modular vaccine development platform, utilizing artificial intelligence to predict optimal antigen combinations, whilst establishing standardized animal models to assess long-term protective efficacy. In addition, chimeric vaccines should also be subject to strict supervision, with a focus on controlling issues such as biosecurity risks, strengthening biosecurity assessment, and preventing potential biological risks caused by genetic recombination. Interdisciplinary integration will become key to overcoming existing technological bottlenecks, providing forward-looking technical reserves to address future emerging pathogenic flavivirus epidemics.

## 8. Conclusions

ISFVs have demonstrated enormous potential as vaccine scaffolds in flavivirus vaccine development. Their unique biological characteristics, similarity to pathogenic flaviviruses, and safety in vertebrates make them an ideal platform for developing novel flavivirus vaccines. Despite facing some challenges, with the deepening research and technological advancements, vaccines based on ISFVs are expected to provide new solutions for preventing and controlling flavivirus infections.

## Figures and Tables

**Table 1:** Comparative characteristics of ISFVs and pathogenic flaviviruses.

Characteristic		Insect-Specific Flavivirus	Pathogenic Flavivirus
Similarity	Genome size	approximately 10.0–11.0 kb (e.g., CFAV is 10.9 kb, Dengue is 10.7 kb)	
	Genome structure	One open reading frame (ORF) encoding 3 structural proteins and 7 non-structural proteins	
	Genome layout	5′UTR-[C-prM-E-NS1-NS2a-NS2b-NS3-NS4a-NS4b-NS5]-3′UTR	
Difference	Host range	Limited to the cells of arthropods such as mosquitoes	Mosquito/tick vectors and mammalian cells
	Replication	Do not replicate in mammalian cells	Do replicate in mammalian cells
	Pathogenicity	Non-pathogenic to vertebrates	Cause serious diseases in humans
	Transmission	Only vertical transmission (mosquito egg transmission)	Spreads horizontally through vector bites

## Data Availability

No new data were created or analyzed in this study. All data supporting the findings of this review are derived from publicly available sources cited throughout the manuscript. The full list of references is provided in the bibliography.
